# Exosomal noncoding RNAs: decoding their role in thyroid cancer progression

**DOI:** 10.3389/fendo.2024.1337226

**Published:** 2024-06-12

**Authors:** Weiming Sun, Chenjun Jiang, Qianqian Liu, Na Wang, Runchun Huang, Gengchen Jiang, Yuxuan Yang

**Affiliations:** ^1^The First Hospital of Lanzhou University, Endocrinology Department, Lanzhou, China; ^2^The First Clinical Medical College, Lanzhou University, Lanzhou, China

**Keywords:** thyroid cancer, exosomes, noncoding RNAs, miRNAs, circRNAs, lncRNAs

## Abstract

Exosomes, as pivotal entities within the tumor microenvironment, orchestrate intercellular communication through the transfer of diverse molecules, among which non-coding RNAs (ncRNAs) such as miRNAs, lncRNAs, and circRNAs play a crucial role. These ncRNAs, endowed with regulatory functions, are selectively incorporated into exosomes. Emerging evidence underscores the significance of exosomal ncRNAs in modulating key oncogenic processes in thyroid cancer (TC), including proliferation, metastasis, epithelial-mesenchymal transition (EMT), angiogenesis, and immunoediting. The unique composition of exosomes shields their cargo from enzymatic and chemical degradation, ensuring their integrity and facilitating their specific expression in plasma. This positions exosomal ncRNAs as promising candidates for novel diagnostic and prognostic biomarkers in TC. Moreover, the potential of exosomes in the therapeutic landscape of TC is increasingly recognized. This review aims to elucidate the intricate relationship between exosomal ncRNAs and TC, fostering a deeper comprehension of their mechanistic involvement. By doing so, it endeavors to propel forward the exploration of exosomal ncRNAs in TC, ultimately paving the way for innovative diagnostic and therapeutic strategies predicated on exosomes and their ncRNA content.

## 1 Introduction

Thyroid cancer (TC), the predominant endocrine malignancy, has seen a consistent rise in incidence across the globe over the past three decades (1). As of 2020, approximately 590,000 individuals worldwide are anticipated to be diagnosed with TC, which manifests with a higher prevalence in women—making it the third most common cancer among women under the age of 50 (2). The United States alone is expected to report around 44,000 new cases in 2022, a figure significantly higher than those of other endocrine cancers, alongside a mortality rate of about 5% (3). This upward trend in TC incidence is multifactorially linked to environmental influences including ionizing radiation and pollution, enhanced dietary factors, and the broader application of advanced screening and diagnostic imaging techniques (2). TC encompasses a spectrum of subtypes categorized based on cellular origin and histopathological characteristics. Over 90% of TC cases are represented by differentiated thyroid cancer (DTC), originating from thyroid follicular cells. DTC is noted for its lower heterogeneity, preserved thyroid tissue morphology, and favorable prognosis, boasting a five-year survival rate exceeding 98% (4). Traditional interventions such as surgery and radioactive iodine therapy have proven effective for most patients with DTC. Conversely, poorly differentiated thyroid cancer (PDTC) and anaplastic thyroid cancer (ATC) represent the more heterogeneous and aggressive TC subtypes, contributing to the majority of TC-related mortalities due to their dismal prognosis and limited treatment options (5). Medullary thyroid carcinoma (MTC), a neuroendocrine tumor arising from parafollicular cells, accounts for about 4% of TC cases and is primarily managed through surgical intervention (6). The treatment landscape for TC has traditionally centered around surgery and radioactive iodine therapy, with the integration of non-invasive approaches like targeted therapy and immunotherapy still under exploration. Particularly, the management of aggressive and metastatic TC variants poses a significant challenge, necessitating a deeper understanding of the molecular underpinnings of TC pathogenesis to innovate treatment modalities. Targeted therapies, including tyrosine kinase inhibitors (TKIs), have emerged over the past decade, offering substantial benefits to patients with advanced TC despite associated adverse effects, thereby driving the search for new therapeutic targets and interventions (7, 8). Amidst these developments, the role of the tumor microenvironment (TME), with a particular focus on exosomes, has gained prominence, heralding new avenues for TC treatment strategies (9).

Exosomes, small extracellular vesicles with a diameter range of 50 nm to 150 nm, were first identified in 1983 in sheep reticulocytes and are predominantly derived from multivesicular bodies (MVBs) (10). These vesicles play a crucial role in intercellular communication, ferrying an array of contents including proteins, lipids, DNA, messenger RNA (mRNA), and more, thereby inducing physiological changes in recipient cells (11). The discovery in 2007 that exosomes contain abundant mRNAs and microRNAs catalyzed a surge in exosomal research (12).

Like exosomes, which were initially regarded as cellular waste, non-coding RNAs (ncRNAs) were once deemed “junk” transcription products. Now recognized as key exosomal contents, ncRNAs encompass a broad class of RNAs that do not translate into proteins but facilitate intercellular regulatory mechanisms within the TME, affecting tumor growth, proliferation, metastasis, angiogenesis, and more (13). Recent studies have illuminated the mechanisms by which exosomal ncRNAs contribute to the onset and progression of TC. This review aims to consolidate current knowledge on exosomes, exosomal ncRNAs, and their interrelation with TC, endeavoring to enrich our understanding of exosomal ncRNAs and propel further research into their mechanistic roles in TC. Ultimately, this work seeks to highlight emerging exosomal ncRNA-related approaches for the targeted therapy of TC.

## 2 Exosomes

### 2.1 Structure and biogenesis of exosomes

**Figure 1:**
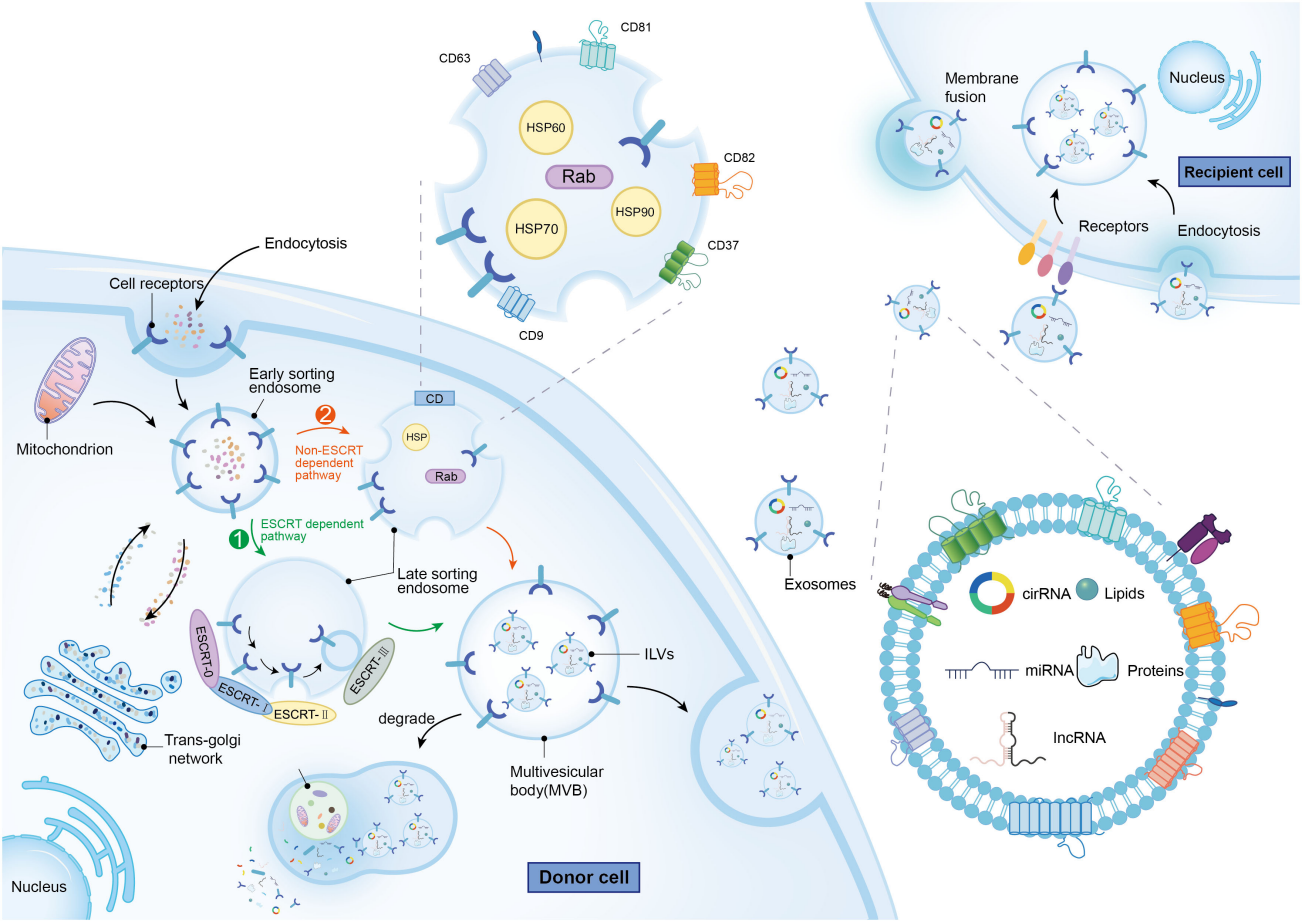
The molecular mechanisms of exosomes biogenesis and metabolism, as well as the characteristics and contents of exosomes. The green arrow represents the ESCRT dependent path, while the orange arrow represents the Non ESCRT dependent path.

Initially regarded as mere cellular waste products, exosomes—ranging in diameter from 50 to 150 nm—are now recognized as vesicles secreted by virtually all cell types to maintain cellular homeostasis. Originating primarily from multivesicular bodies (MVBs), exosomes are found in all bodily fluids including plasma, serum, lymph, digestive fluids, urine, breast milk, cerebrospinal fluid, amniotic fluid, and semen (14). Distinct from microparticles (100–1000 nm in diameter) and apoptotic vesicles (1–4 μm in diameter), which also derive from MVBs, exosomes are generated through a unique process. This process involves the outward budding of the plasma membrane, leading to the formation of intraluminal vesicles (ILVs) within MVBs (15). Exosome biogenesis involves two main, partially overlapping mechanisms (**Figure 1**). Both pathways initiate with the formation of early secretory endosomes through endocytosis of the cytoplasmic membrane. These early endosomes then generate intraluminal vesicles (ILVs) via endogenous budding. The resulting structure, known as a multivesicular body (MVB), ranges from 100 to 250 nm in diameter and encapsulates multiple ILVs of 50 to 150 nm in diameter. The MVB undergoes acidification to mature into a late endosome (16). One of the biogenesis pathways, the ESCRT-dependent pathway, involves the endosomal sorting complex required for transport (ESCRT) proteins. This pathway starts when ESCRT-0 recognizes ubiquitylated cargoes, initiating the ESCRT process. Subsequently, ESCRT-I and ESCRT-II bind to each other, forming a cargo-rich region, while ESCRT-III packages the contents into mature vesicles through a mechanism involving deubiquitylation and the invagination of the MVB to form ILVs (17). In contrast, the non-ESCRT-dependent pathway relies on chaperone proteins such as HSP60, HSP70, and HSP90, four-transmembrane proteins including CD63, CD81, CD82, CD37, and CD9, as well as the Rab-associated protein family (18). The precise mechanisms determining the metabolic fate of MVBs remain elusive; however, it is known that MVBs enriched in cholesterol may fuse with the cell membrane and release their contents externally, while others are more likely to undergo degradation (19).

### 2.2 Composition and biological function of exosomes

Exosomes are complex entities primarily composed of lipids, proteins, and nucleic acids. The outer structure is predominantly a lipid bilayer comprising phosphatidylcholine (PC), phosphatidylethanolamine (PE), phosphatidylinositol (PI), phosphatidylserine (PS), and sphingomyelin. The stability and functional properties of exosomes are largely dictated by the content and composition of these lipid molecules (20). Proteins within exosomes are situated in phospholipid domains known as lipid rafts and may also be encapsulated within the membrane. These proteins are categorized into two groups: non-specific proteins, common across all exosomal types and often used as markers of exocytosis (e.g., CD9, CD63, CD81, HSP70), and specific proteins, which vary depending on the origin of the parent cell and include markers such as MHC II in B-lymphocyte exosomes (21), and Granzyme and Perforin in T lymphocyte exosomes (22). Exosomes also contain a diverse array of RNAs with biological functions, ranging from mRNAs with translational capabilities to non-coding RNAs (ncRNAs) with regulatory roles. Significant research emphasis has been placed on ncRNAs within exosomes, including miRNAs, circRNAs, and lncRNAs, due to their potential to modulate the tumor microenvironment. These ncRNAs are implicated in critical oncogenic processes such as tumor growth, proliferation, metastasis, angiogenesis, drug resistance, and immune evasion (23). Additionally, exosomes play a role in cancer cell metabolism. For instance, in lung cancer, exosomal miR-3679–5p downregulates NEDD4L, consequently increasing c-Myc activity which enhances glycolysis—a vital process for the proliferation of cancer cells (24). Furthermore, exosomes influence cancer migration and invasion. In lung cancer, miRNA-204 targets the KLF7-Akt/HIF-1α axis, thereby inhibiting epithelial-mesenchymal transition (EMT) (25). In the context of angiogenesis, the expression of circSHKBP1 in extracellular vesicles from gastric cancer patients has been shown to promote angiogenesis by upregulating HUR and VEGF via the sponge mechanism of miR-582–3p (26). Additionally, the immune response in cancer is also modulated by exosomes. For example, in hepatocellular carcinoma, exosomal circCCAR1 taken up by CD8+ T cells promotes CD8+ T cell dysfunction and resistance to PD-1 inhibitors (27).

### 2.3 NcRNA in exosomes

**Figure 2:**
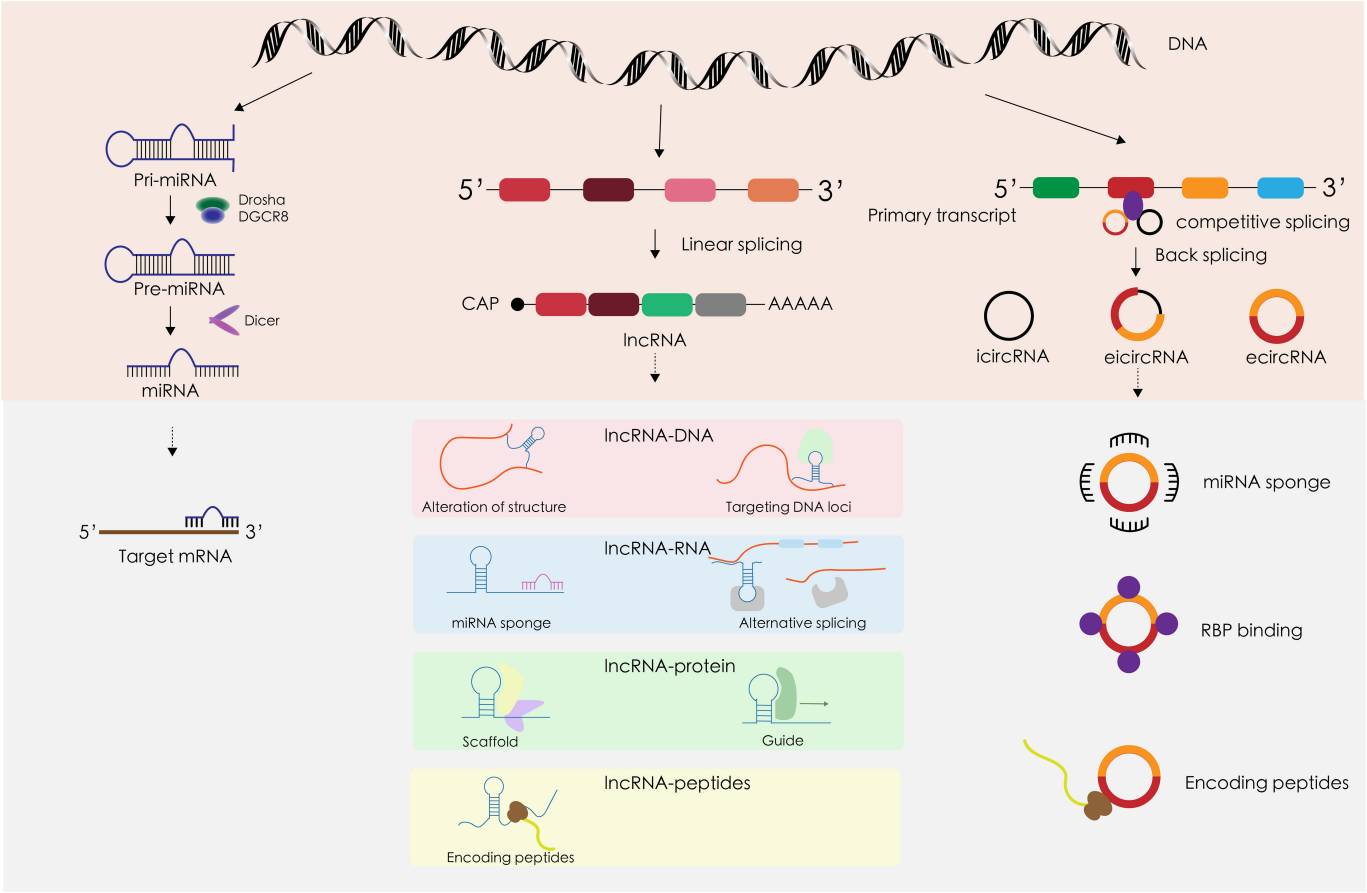
The structural characteristics, occurrence and development, and mechanism of action in TC of miRNA, lncRNA, and circRNA.

Approximately 1.22% of the human genome comprises protein-coding genes, yet over 80% is transcribed into non-coding RNAs (ncRNAs) such as miRNAs and lncRNAs. The functions of most ncRNAs are still under investigation (28). Initially considered transcriptional noise, ncRNAs have been recognized in recent decades for their significant roles in various diseases, particularly cancer (29). This discussion will concentrate on three prominent types of ncRNAs—miRNA, lncRNA, and circRNA—focusing on their biogenesis, structure, and mechanisms of action (**Figure 2**). MicroRNAs (miRNAs) are a well-studied class of small ncRNAs about 18–25 nucleotides long. They primarily regulate gene expression by targeting the 3’ non-coding regions of mRNAs, influencing mRNA translation (30). Long non-coding RNAs (lncRNAs), which represent a large portion of eukaryotic ncRNAs, are typically longer than 200 nucleotides. However, many lncRNAs have yet to be fully characterized (31). lncRNAs modulate gene expression post-transcriptionally through various mechanisms, including serving as miRNA sponges and binding to proteins (32). Circular RNAs (circRNAs) form a novel class of endogenous ncRNAs. Unlike linear RNAs, circRNAs have a unique covalent closed-loop structure. They participate in numerous biological processes in both physiological and pathological states, similar to lncRNAs, by acting as miRNA sponges and interacting with proteins (33).

#### 2.3.1 MiRNA

MiRNA biosynthesis initiates in the nucleus, where DNA is transcribed into primary miRNAs (pri-miRNAs) that can be several thousand bases long. This transcription is facilitated by RNA polymerase II. The pri-miRNAs are then processed into precursor miRNAs (pre-miRNAs) by the DGCR8-Drosha complex. These pre-miRNAs are exported from the nucleus by the transport protein Exportin5. In the cytoplasm, pre-miRNAs are further processed into single-stranded miRNAs by the enzyme Dicer. Mature miRNAs are subsequently sorted into exosomes through various mechanisms (34). The principal gene regulatory function of miRNAs involves post-transcriptional gene silencing, mediated by the miRNA-induced silencing complex (miRISC). The classical miRISC mechanism involves three key steps mediated by miRNAs, Argonaute 2 (Ago 2), and the trinucleotide repeat-containing gene 6 (TNRC6) (35): (1) Ago2 recruits TNRC6, which subsequently recruits the CCR4-NOT de-adenylase complex, leading to de-adenylation and degradation of mRNA. (2) TNRC6 recruits the Dcp 1/2 decapitation complex, which cleaves the 5’ cap of mRNA and reduces mRNA stability. (3) With the binding of Ago 2, the mRNA is unable to bind to the ribosome, thus inhibiting translation. MiRNAs are frequently dysregulated in malignant tumors, playing a critical role in cancer development (36). This dysregulation varies across different types of tumors, with miRNAs such as miR-221, miR-222, miR-146b, and miR-155 commonly found to be aberrant (37). Notably, miR-221 and miR-222 are overexpressed in several cancers including gastrointestinal, hepatocellular, and papillary thyroid cancers, where they target and suppress tumor suppressor genes (38). The biological behavior of tumors is significantly influenced by the tumor microenvironment (39). As pivotal communicators within this microenvironment, exosomal miRNAs not only mediate and regulate the growth and proliferation of tumor cells but also impact vascular endothelial and immune cells, affecting processes such as immune evasion and angiogenesis (34). For instance, exosomal miR-21–5p is markedly upregulated in papillary thyroid carcinoma (PTC) and enhances angiogenesis in human umbilical vein endothelial cells (HUVECs) (40). Additionally, in non-small cell lung cancer, exosomal miR-21 and miR-29a interact with Toll-like receptors (TLRs) to provoke pre-metastatic inflammation, thereby facilitating tumor metastasis (41).

#### 2.3.2 LncRNAs

Long non-coding RNAs (lncRNAs) share several transcriptional and processing similarities with mRNAs, such as being transcribed by RNA polymerase II from chromatin-associated promoter regions, and undergoing splicing, 5’ capping, and 3’ polyadenylation. These similarities underscore that lncRNAs follow a transcription and processing pathway akin to that of mRNAs. However, lncRNAs often exhibit more specific expression patterns compared to mRNAs, possibly due to more stringent regulatory mechanisms (42). Additionally, lncRNAs may contain sequence motifs that recruit nuclear factors, thereby influencing nuclear localization (43). Many lncRNAs are exported to the cytoplasm and have been identified in the exosomes of human blood, although the mechanisms governing their sorting remain elusive and are likely related to interactions between specific lncRNA sequences and RNA-binding proteins (44). The highly specific expression of exosomal lncRNAs makes them significant discriminatory markers for various diseases, including cancer. Exosomal miRNA expression varies in different types of cancer and at different stages of a certain cancer, which makes miRNAs important markers for cancer diagnosis and prognosis, but little overlap has been reported in relatively similar findings in a given cancer (45). As the potential of lncRNAs as stable serum biomarkers gains recognition, their utility as cancer biomarkers is increasingly discussed (46). Unlike the relatively homogeneous regulatory patterns of miRNAs, lncRNAs interact with DNA, RNA, and proteins at multiple regulatory levels. These interactions include modifying chromatin structure, interfering with transcription, and regulating gene expression at transcriptional, post-transcriptional, translational, and post-translational stages (43). Similarly, exosomal lncRNAs, which are key components of the tumor microenvironment, play critical roles in cancer progression, mirroring the multifaceted functions of exosomal miRNAs. These roles encompass promoting tumor proliferation, metastasis, angiogenesis, immune escape, drug resistance, and more (47, 48).

#### 2.3.3 CircRNAs

Circular RNAs (circRNAs) are a class of non-coding RNAs characterized by their unique closed-loop structure. They are produced through reverse splicing that connects the downstream 5’ splice site to the upstream 3’ splice site in reverse order, competing with conventional linear splicing (49). CircRNAs are classified into three types based on their sequence content: exonic circRNAs (EcRNAs), intronic circRNAs (CiRNAs), and exon-intronic circRNAs (EIcRNAs) (50). CiRNAs generally localize to the nucleus, while EcRNAs, often modified by m6A, are exported to the cytoplasm (51). In recent years, circRNAs have also been identified as components of exosomes. Unlike linear RNAs such as miRNAs and lncRNAs, circRNAs lack a 5’ cap and a 3’ tail, contributing to their high stability and resistance to ribonucleases (52). This enhanced stability positions circRNAs as promising biomarkers and targeted drug carriers for various cancers. CircRNAs exert their biological effects through several mechanisms: (1) MiRNA Sponges: CircRNAs can sequester miRNAs, acting as competing endogenous RNAs to decrease their biological activity and indirectly upregulate the activity of miRNA target genes (53). (2) Protein Interaction: Certain circRNAs have specific protein-binding sites that can isolate proteins, acting as sponge proteins, or serve as protein scaffolds to facilitate the action of specific proteins (49, 50). (3) Translation into Proteins: Recent studies suggest that circRNAs can initiate translation through cap-independent mechanisms, primarily internal ribosome entry sites (IRES) and m6A-driven processes, thereby directly influencing cellular functions (54). (4) Regulation of transcription: The reverse splicing of circRNAs can compete with mRNA splicing of the same gene, affecting gene expression (49). CiRNAs and EIciRNAs can interact with RNA Polymerase II to enhance gene transcription (50). As components of the tumor microenvironment, exosomal circRNAs share functional similarities with other non-coding RNAs in regulating critical oncogenic processes such as tumor growth, proliferation, metastasis, epithelial-mesenchymal transition (EMT), and angiogenesis (55).

## 3 Role of exosomes and exosomal ncRNAs in TC progression

**Figure 3:**
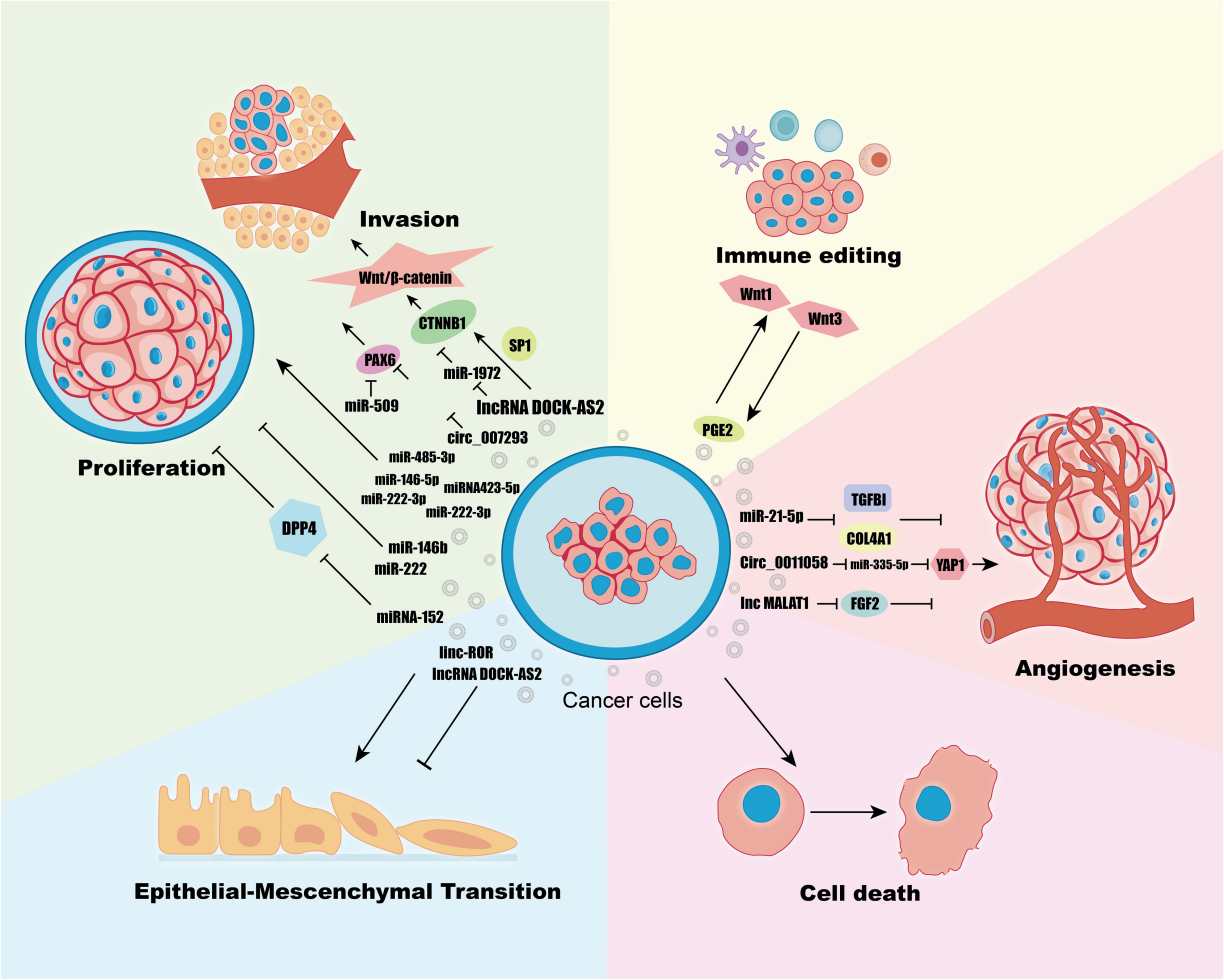
Regulation of Proliferation, Invasion, Immune Editing, Angiogenesis, Cell Death, EMT in TC Cells by Exosomes ncRNAs.

The tumor microenvironment (TME) is predominantly composed of the extracellular matrix (ECM), stromal cells—including fibroblasts, myofibroblasts, mesenchymal stem cells, neuroendocrine cells, adipocytes, vascular endothelial cells, and the lymphatic vascular network—and various immune cells such as tumor-infiltrating lymphocytes (T and B lymphocytes), natural killer cells, and tumor-associated macrophages (56). As tumors evolve, their ecological niche, the TME, becomes increasingly heterogeneous, reflecting a complex interplay that significantly influences tumor initiation and progression (57). A key question arises: within the TME, which component is pivotal in linking various cells and facilitating signal transmission? To address this, we will explore the integral role of exosomal noncoding RNAs. These RNAs crucially impact tumor behavior in several contexts, including proliferation, metastasis, epithelial-mesenchymal transition (EMT), angiogenesis, immunoediting, and cell death (58) (**Figure 3**) (**Table 1**).

**Table 1:** Changes in ncRNAs involved in exosomes in thyroid cancer and other cancers.

ncRNA	Alteration	functions in TC	Cancer and/or other disorders
lncRNA DOCK9-AS2	Up	Proliferation(+) Stemness(+) Migration(+) Invasion(+)	
hsa_circ_007293	Up	Invasion(+) EMT(+) Migration(+)	Bladder cancer Gastric cancer
miR-485–3p	Up	Drug sensitivity(-)	Colorectal cancer Breast cancer
miR-25–3p, miR-296–5p, miR-92a-3p,	Up	Biomarker	Colorectal cancer Breast cancer Hepatocellular carcinoma
miR-145	Down	Proliferation(-) Migration(-)	Ovarian carcinoma Gastric cancer Breast cancer
miR-21–5p	Up	Angiogenesis(+)	Gastric cancer Colorectal cancer Hepatocellular carcinoma
linc-ROR	Up	EMT(+)	Breast cancer Pancreatic cancer
microRNA-152	Down	Proliferation(-) Migration(-) Invasion(-)	Hepatocellular carcinoma Bladder cancer
miR-5189–3p	Up	Biomarker	Breast cancer
miR24–3p, miR146a-5p, miR181a-5p, miR382–5p		Biomarker	
miR-6774–3p, miR-6879–5p	Up	Biomarker	
miR-346, miR-10a-5p, miR-34a-5p	Up	Biomarker	Prostate cancer Colorectal cancer
miRNA423–5p	Up	Migration(+) Invasion(+)	Gastric cancer
hsacirc_007293, hsacirc_031752, hsacirc_020135	Up	Biomarker	
SCD-1 siRNA	Down	Apoptotic(+)	
miR-29a	Down	Proliferation(-) Migration(-) Invasion(-)	Breast cancer Prostate cancer
miR-146b, miR-222	Up	Biomarker	
hsa-miR-129–2, hsa-miR-889		Biomarker	
miR-148a-3p		Biomarker	
miR-30c-5p	Down	Proliferation(-) Migration(-)	
miRNA let-7	Up	Biomarker	
miR-146b-5p, miR-21a-5p	Up	Biomarker	
miR-221–3p	Up	Proliferation(+) EMT(+) Migration(+) Invasion(+)	Breast cancer Lung cancer

+ means promoting, − means inhibiting.

### 3.1 Proliferation and metastasis in TC

Proliferation and metastasis are fundamental malignant behaviors in cancer, where tumors create a conducive tumor microenvironment (TME) that mediates communication between tumor cells and stromal cells through various biologically active substances, including ncRNAs, proteins, and DNA delivered by exosomes (59). For instance, lncRNA DOCK-AS2 is upregulated in papillary thyroid carcinoma (PTC) and notably elevated in metastatic patients. It is highly expressed in exosomes derived from PTC cancer stem cells (CSCs) and can be transferred to recipient PTC cells via exosomes, promoting their proliferation, migration, and invasion (60). The Wnt/β-catenin signaling pathway, known to regulate cell proliferation and metastasis, is activated by DOCK9-AS2 through two mechanisms (61): (1) It regulates CTNNB1 expression and activates the Wnt/β-catenin pathway by inducing CTNNB1 promoter activation in cooperation with SP1. (2) It upregulates CTNNB1 expression and activates the Wnt/β-catenin pathway through miR-1972 sponging. Another study on PTC (62) revealed enrichment of hsa_circ_007293 in exosomes derived from sera of PTC patients and PTC cell lines, and it was shown that the exosome hsa_circ_007293 promotes proliferation, migration, and invasion of TPC-1 and KTC-1 thyroid cancer cell lines, and knockdown of hsa_circ_007293 inhibited this process. The PAX family of PAX6’s role as an oncogene has been demonstrated in a variety of cancers (63). The mechanism of action of hsa_circ_007293 is to act as a ceRNA to promote PAX6 expression by competitively binding miR-653–5p (62). MiR-509, which is down-regulated in PTC, has also been found to target PAX6 thereby inhibiting tumor proliferation and invasion (64). Exosomal miR-485–3p is upregulated in PTC and associated with larger tumors (≥1cm), advanced clinical stage, extrathyroidal extension, BRAF mutations, and lymph node metastasis (65). The specific role of miR-485–3p in PTC and its molecular mechanisms require further elucidation. Other studies have linked various miRNAs, including miR-181a-5p, miR-376a-3p, miR-382–5p, miR-146b-5p, and miR-222–3p, with lymph node metastasis, showing enhanced migration and invasion in PTC cells when upregulated in exosomes (66, 67). Among them, miR-146–5p and miR-222–3p, which were upregulated in exosomes, significantly enhanced the migration and invasion of PTC cells (67). Another study showed that exosomal miRNA423–5p was upregulated in PTC patients, and overexpression of miRNA423–5p mimics derived from TPC-1 cells was verified to promote migration and invasion of PTC cells (68). Exosomes miR-146b and miR-222 are overexpressed in exosomes secreted by PTC cell lines and have negative proliferative effects on PTC cell lines and normal thyroid tissue cell lines (69).

Mesenchymal stem cells (MSCs), pluripotent stem cells capable of differentiating into adipocytes, osteoblasts, and chondrocytes, play a significant role in the TME (70). Exosomal miRNA-152 from MSCs has been shown to inhibit proliferation, migration, and invasion of thyroid carcinoma cells through interaction with DPP4 (71). However, the role of MSCs is not invariable; tumor cells can regulate the TME through their own release of exosomes, altering the extracellular matrix, changing the stromal cell phenotype to a metastatic phenotype, and facilitating the formation of pre-metastatic ecological niches for tumor progression, e.g., tumor cells can reprogram MSCs in the TME into cancer-associated fibroblasts (CAFs) for tumor development through cell-to-cell communication, especially through the release of exosomes (72). Affected CAFs can also promote tumor progression by secreting exosomal ncRNAs that deliver signaling molecules to tumor cells, and these aforementioned interactions between tumor cells and CAFs have been revealed in many cancers (73, 74). However, the effects of multiple interactions between TC cells and exosomal ncRNAs and TME on these mechanisms need to be further investigated.

### 3.2 EMT in TC

Epithelial-mesenchymal transition (EMT) is a reversible cellular program where epithelial cells acquire a mesenchymal phenotype, transitioning from a cobblestone shape to a spindle-shaped morphology. This process is reversible under certain conditions through mesenchymal-epithelial transition (MET). Both EMT and MET are critical during normal development and cancer progression (75). Most malignant progressions in cancer are associated with EMT activation in tumor cells (76, 77). In the tumor microenvironment (TME), epithelial cells receive signals that induce EMT by activating downstream signaling pathways. Key characteristics of EMT include the acquisition of stem cell properties, decreased adhesion, increased invasiveness, enhanced drug resistance, and immune evasion (75, 78). It has been observed that cancer stem cells (CSCs) are closely linked with EMT. Signals from cancer-associated fibroblasts (CAFs), T-lymphocytes, and tumor-associated macrophages (TAMs) within the TME induce EMT, promoting tumor cell dedifferentiation and metabolic redistribution, thus generating CSCs in an intermediate epithelial-mesenchymal state (79). In thyroid cancer, CSC-derived exosomes containing lncRNAs such as MALAT1 and linc-ROR, along with transcription factors like SOX2 and SLUG, promote proliferation and induce EMT in normal thyroid cells—specifically when exosomes contain linc-ROR (80). Previous research also demonstrates that exosomal lncRNA DOCK9-AS2 from CSCs downregulates EMT markers such as E-calmodulin, N-calmodulin, and MMP, and activates EMT via the Wnt/β-catenin pathway (60). Similarly, exosome-enriched circ007293 promotes EMT activation in PTC cells by downregulating miR-653–5p to induce PAX6 expression (62). In addition to this, miRNAs have been shown to regulate the expression of EMT markers and modulate multiple signaling pathways that induce EMT (81). Moreover, miRNAs like miR-145, found down-regulated in thyroid cancer tissues and secreted into serum via exosomes, have shown potential in regulating EMT. Overexpression of miR-145 inhibits cell invasion and upregulates N-calmodulin, while its downregulation in tumor tissues promotes tumor growth and metastasis by inducing EMT (82). Hypoxic PTC cell-derived exosomes containing overexpressed miR-221–3p targeted inhibition of ZFAND5 expression, enhancing invasion and EMT in normal PTC cells (83). In addition, there are many ncRNAs that have been validated to induce EMT in TC, but the study of the role of exosome-derived ncRNAs in driving EMT is incomplete, and the role of TC-derived exosomes in EMT needs further investigation. EMT in tumors is not a binary state: EMT is indicated only by epithelial-mesenchymal markers such as E-calmodulin, waveform protein or other EMT transcription factors, morphological changes (84). It has been increasingly demonstrated that EMT is in fact a hybrid epithelial/mesenchymal (hybrid E/M) “profile”, and that dynamic changes in the EMT profile under different environmental stresses are closely related to cellular plasticity, metastatic potential, and so on (85). In particular, recent studies have found that mixed EMT has a higher probability of obtaining stem cells, and epithelial-mesenchymal plasticity (EMP) as a new concept implies that EMT does not necessarily manifest as a binary transition between the two extremes, but rather that it can enable cells to become mixed phenotypes with both epithelial and mesenchymal characteristics, such that epithelial cells enter the stem cell state primarily through EMT and highly mesenchymal fibroblasts enter the stem cell state primarily through MET, respectively (86). The dynamic balance of EMT/MET activation seems to be more important nowadays. Whereas exosomal ncRNAs, as important signaling regulatory molecules in cellular communication, are involved in the activation of EMT in TC, we therefore hypothesized that exosomal ncRNAs would also be involved in regulating the change of epithelial-mesenchymal dynamics equilibrium. Therefore, targeting the inhibition of EMT or regulation of epithelial-mesenchymal dynamics through exosomal delivery of ncRNAs, proteins, etc. in TC may have potential research significance.

### 3.3 Angiogenesis in TC

Angiogenesis, the development and formation of new blood vessels from pre-existing ones, is typically quiescent under normal physiological conditions but plays a crucial role in the growth, invasion, and metastasis of neoplastic tumors, serving as an initial marker of cancer (87). In the tumor microenvironment, angiogenesis occurs through three primary mechanisms: (1) Sprouting of new blood vessels from mature, pre-existing vessels. (2) Neovascularization initiated by the development and maturation of endothelial precursor cells, known as angioblasts. (3) Formation of new capillaries by tumor cells or other non-endothelial cells without direct support from vascular endothelial cells (88). Neovascularization is characterized by structural abnormalities, high wall permeability, and irregular distribution (89). Many recent studies have shown that tumor cell-derived exosomes contain pro-vascular angiogenic signaling molecules such as proteins and RNAs, and that the generation of new blood vessels depends on the level of exosomes generated by tumor cells in the early stages of tumor production (90). Hypoxia is an important feature of the tumor microenvironment, and studies have shown that the exosome miR-21–5p secreted from PTC in hypoxic environments enhances the tube formation and migration of human umbilical vein endothelial cells (HUVEC). MiR-21–5p is delivered as an exosome between the tumor cells and the endothelial cells. TGFBI, COL4A1 have been shown in previous studies to be anti-angiogenic factors, miR-21–5p targeted and inhibited the expression of TGFBI, COL4A1 in PTC endothelial cells thereby promoting angiogenesis (40). Circ_0011058 expression was abnormally elevated in PTC tissues and cells, and Circ_0011058 upregulated YAP1 by acting as a miR-335–5p sponge to promote PTC cell proliferation, angiogenesis (91). Another study showed that lnc MALAT1 in TAM inhibited the release of inflammatory cytokines from FGF2 protein, promoted FTC cell proliferation, migration and invasion, and induced angiogenesis (92). If the tumor cells are unsupported by blood vessels, the size of the tumor eventually stays at only a few millimeters, whereas when the tumor is transferred to mice with a vascular system, it can rapidly grow to a size of several centimeters (93). Tumor angiogenesis increases the number of blood vessels and the permeability of the vessel wall, providing sufficient nutrients for tumor growth and conditions for subsequent metastasis (94). The above studies suggest that exosomes can directly target angiogenesis as carriers of anticancer drugs. In addition to this. exosomes play an indirect role in antiangiogenic therapy. For example, LncARSR increases resistance to the anti-angiogenic drug sunitinib in kidney cancer cells via exosomes (95). RAB27B activates the VEGF pro-angiogenic pathway in multiple cancers and may serve as a marker of sunitinib sensitivity (96). In summary, exosomal ncRNAs play an important role in promoting TC angiogenesis and are potential therapeutic targets.

### 3.4 Immunoediting in TC

Immunoediting refers to the dual role of the immune system on tumors (97). According to the dual role can be divided into three stages of immune editing in tumor development: (1) elimination stage: tumor cells with antigenic properties are recognized and eliminated and killed by immune cells; (2) equilibrium stage: genetic and epigenetic changes, tumor cells mutate and evolve their antigenic properties to be decreased or defective and induce immunosuppression; (3) escape stage: this stage has obvious clinical symptoms, the immune system is unable to limit the growth of tumors, the reduction of the ability to immunorecognition, the increase of the resistance of tumor cells, and the formation of the immune suppression tumor microenvironment promote the immune escape of the tumor (98, 99). Exosomes, as a continuous mediator of communication between tumor tissues and the immune system in the tumor immune microenvironment, have different characteristics of their roles at different stages, with the escape stage being the most studied (100). Tumor-derived exosomes deliver transforming growth factor-β (TGF-β) to inhibit DC differentiation and maturation (101). Binding of programmed death ligand (PD-L1) to its receptor inhibits T-cell activation, and overexpressed PD-L1 is found in exosomes in many cancers (102). TAMs, an important component of the tumor immune microenvironment, are divided into inflammatory macrophages (M1) and anti-inflammatory macrophages (M2), with the M2 phenotype favoring immunosuppression (103). Exosomal ncRNA induces polarization of TAMs to M2 phenotype in ovarian and hepatocellular carcinoma (104, 105). In TC, the density of TAM in the microenvironment was positively correlated with TC progression (106). TC cells and M2-TAMs can promote each other, and M2-TAMs promote TC de-differentiation, proliferation, and metastasis by activating the Wnt signaling pathway through the secretion of Wnt1 and Wnt3 ligands (107). TC cells secrete PGE2 to promote M2 polarization (108). It is hypothesized that exosomal ncRNAs play equally important roles in these processes described above, but this hypothesis requires further study. Natural killer (NK) cells, which are also part of the tumor immune microenvironment, do not need to rely on MHC molecules to play a role in immune regulation, and as important sentinels of the immune surveillance system, NK cells can recognize malignant cells very early and resist tumor progression and metastasis without additional activation (109). The role of tumor-derived exosomal ncRNAs in weakening NK cells in a variety of cancers has been demonstrated. For example, miR-92b attenuates NK cell activity in hepatocellular carcinoma (110). CircUHRF1 decreases the percentage of NK cell infiltration in hepatocellular carcinoma (111). LncRNA SNHG10 inhibits NK cytotoxicity in colorectal cancer (112) and so on. In addition, extracellular vesicles from the NK cell line NK3.3 were found to have a broad spectrum of antitumor effects (113). And exosome mimics from NK cells are even more potent anti-tumor effects in cancers including TCs (114). The use of NK cell immunotherapy for the treatment of solid cancers has been heavily researched in recent years and has emerged as a promising therapeutic approach for the treatment of solid tumors and hematologic malignancies (115). Exosomes from tumor cells exert immunosuppressive effects on immune cells such as T cells, DC cells, NK cells, TAM, etc. Therefore, targeted inhibition of tumor cell exosomes and exosomal RNA has become a new type of immunotherapy. Heat shock proteins, one of the cargoes of exosomes, are involved in the immune response of tumor cells, and the levels of Hsp27, Hsp60, and Hsp90 were elevated in TPC tissues compared with normal peritumoral tissues and benign goiter. The levels of HSPs in the exosomes of TPC patients before surgery were significantly higher than those in the exosomes of the same patients and benign goiter patients after surgery (116). It can therefore be inferred that exosomal ncRNAs likewise have a potential biomarker role. TC is one of the most immunogenic tumors (117). Further expansion of the molecular mechanisms of exosomal ncRNAs in TC immunoediting, and studies on exosomal changes at earlier stages of immunoediting will make emerging immunotherapies regarding exosomes promising TC treatments.

### 3.5 Cell death in TC

Cell death can be broadly categorized into accidental cell death (ACD) and regulatory cell death (RCD) according to whether it is affected by drugs and gene regulation, and RCD is mediated by well-defined molecular mechanisms and plays an important role in maintaining homeostasis in multicellular organisms, and in disease progression (118). RCD also known as programmed death (PCD) can be subdivided into apoptosis, autophagy, iron death, pyroptosis, and necroptosis. Cancers often exhibit resistance to apoptosis, and loss of apoptosis can lead to uncontrolled cell proliferation (119). Anti-tumor therapies targeting apoptosis have made remarkable achievements in the last three decades, such as vitamin C promotes apoptosis and cell cycle arrest in aggressive thyroid cancer cells by attenuating the feedback activation of the MAPK/ERK as well as the PI3K/AKT pathways in PLX4032 (120). Picrasidine exerts antitumor effects by inhibiting PTC growth through down-regulation of miR-182–5p expression inducing apoptosis in PTC cells (121). The involvement of exosomal ncRNAs in the regulation of tumor apoptosis also contributes to pro-apoptotic therapies, e.g., exosomes produced by hypoxic PTC cells and miR-221–3p in exosomes inhibit apoptosis in normal PTC cells *in vitro* (83). Exosomal circPACRGL targeting miR-142–3p/miR-506–3p overexpression of TGF-β1 inhibits apoptosis in colorectal cancer cells (122). CAFs-derived exosomes enriched with miR-93–5p acted on CRC cells to down-regulate FOXA1 and up-regulate TGFB3, which ultimately inhibited the nuclear accumulation of TGFB3 and suppressed radiation-induced apoptosis in CRC cells (123). Pro-apoptotic therapies as one of the cancer treatments have been limited due to their resistance, which has been explained by various reasons, such as disruption of apoptotic mechanisms, increased expression of therapeutic targets, and high heterogeneity of tumor cells (124). Therefore, some therapeutic strategies to bypass apoptosis or non-apoptotic PCD are nowadays hotspots for cancer treatment. In general, autophagy, iron death, pyroptosis, and necroptosis, which are non-apoptotic PCDs, exert anti-tumor effects, e.g., autophagy degrades intracellular toxic substances and secretes signaling molecules such as proteins, hormones, etc., in an autophagy-mediated manner (125). Malignant tumors usually exhibit increased secretion of exosomes, and it has been shown that inhibition of autophagy can either promote or inhibit the release of exosomes, depending largely on the environment, and that the amount and content of exosomes are also influenced by autophagy (126). Activation of NF-κB signaling pathway by exosomal miR-1910–3p targeting MTMR3 promotes autophagy in breast cancer cells (127). In addition, modulation of autophagy activity can enhance the effects of many antitumor drugs, and a series of exosomal miRNAs can modulate drug resistance by regulating autophagy, e.g., trastuzumab-resistant cells in breast cancer exhibit enhanced autophagy activity that can be inhibited by miR-567 from exosomes, resulting in an increase in sensitivity (128). In addition to autophagy, cellular pyroptosis, iron death, and necrotic apoptosis can exert synergistic anti-tumor immune responses, and tumor cells can release signals to recruit CD8+ T cells through cellular pyroptosis, while on the other hand, CD8+ T cells induce tumor cell pyroptosis through the GSDME-GZMB axis (129). Fibroblasts in TME can induce a strong anti-tumor immune response through necrotic apoptosis with NF-κB signaling pathway (130). However, non-apoptotic PCD can also exhibit antagonistic tumor immune responses, e.g., MHC complexes on the surface of cancer cells and immune cells can be degraded by autophagy for immune evasion (131). Most immune cells, including CD8+ T cells, NK cells, and DC cells, are sensitive to iron death, and induction of iron death in these immune cells by GPX4 inhibitors decreases their immunocidal function (132). It is not difficult to see that the relationship between non-apoptotic PCD and tumor immunity is complex, not only because they play a synergistic anti-tumor immune role as well as an antagonistic tumor immune response, and because different types of TME cells play different roles in this. Targeting non-apoptotic PCD is a promising strategy for assisting immunotherapy, but DAMP produced by inducing non-apoptotic PCD can also stimulate normal cell death, the upstream mechanisms common to the various non-apoptotic PCDs are poorly investigated, and the exact molecular mechanisms that lead to cell death are not yet fully understood. Molecular events occurring downstream of lipid peroxidation in iron death, for example (133). In lung adenocarcinoma, exosomal circRNA_101093 interacts with and increases fatty acid binding protein 3 (FABP3), a polyunsaturated fatty acid essential for the increased plasma membrane peroxidation associated with iron death, FABP3 transports AA and promotes its reaction with taurine, and AA decreases, preventing AA adulteration of the plasma membrane desensitizing LUAD cells to iron death (134). In gastric cancer, CAF secretes the exosome miR-522, which targets and inhibits arachidonic acid lipoxygenase 15 (ALOX15), thereby blocking the accumulation of lipid ROS and inhibiting iron death in cancer cells (135). We predict that ncRNAs, which are involved as important signaling molecules and biomarkers as important players, may become more specific potential therapeutic targets, providing new directions and perspectives for future research, while exosomes, as a new type of nanocarrier, may also be able to target specific cells more precisely.

## 4 Potential clinical applications of exosomes and exosomal ncRNAs in TCs

### 4.1 Promising biomarkers

**Figure 4:**
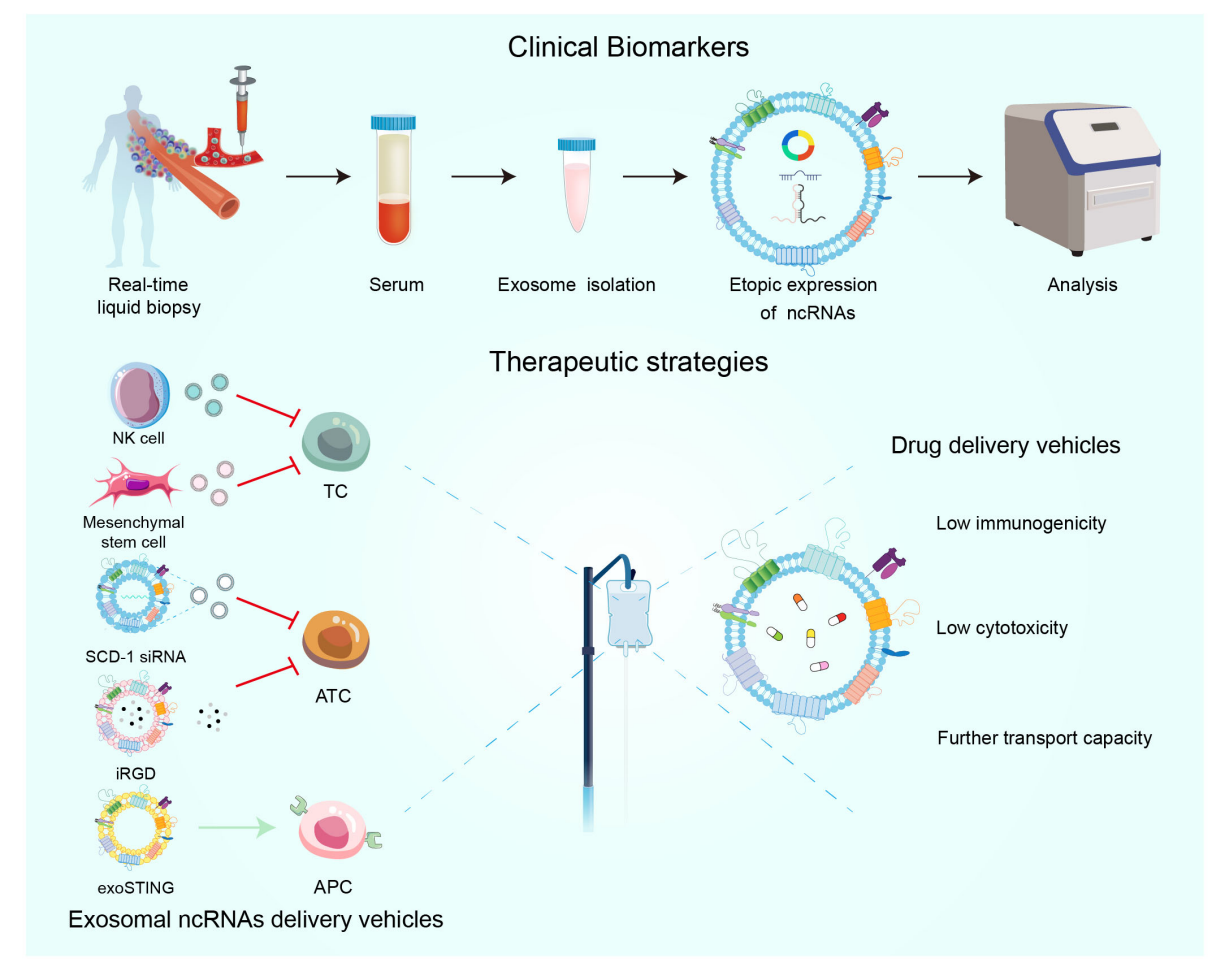
The significance of extracellular vesicles ncRNAs as biomarkers in the diagnosis and prognosis of TC. Emerging therapeutic methods for extracellular vesicles in TC.

Fine needle aspiration biopsy (FNAB) is the gold standard for the initial diagnosis of TC, but it still has a high incidence of non-diagnostic findings (136). Under such a trend, liquid biopsy has entered the field of vision as a new diagnostic method with the advantages of low trauma, easy collection, and dynamic analysis, and its candidate markers include circulating tumor cells, circulating free nucleic acids, exosomes, etc. (137) (**Figure 4**). Recently, exosome-derived ncRNA has attracted increasing attention as a diagnostic indicator for liquid biopsy. How to distinguish malignant follicular thyroid carcinoma (FTC) from benign follicular adenoma (FA), which cannot be fully discriminated even by FNAB and ultrasonography (138). Whereas miRNA let-7 in exosomes from TPO(+) cells is significantly overexpressed in the serum of FTC patients, which provides a new marker for liquid biopsy in FTC (139). Plasma exosomes miR-146b-5p and miR-21a-5p differ significantly in abundance between patients with PTC and benign tumors and have the potential to be diagnostic biomarkers for PTC (140). A study showed 129 differentially expressed miRNAs in 13 PTC patients and 7 NG patients, with miR-5189–3p being the most diagnostic value (141). In another study, four exosomal miRNAs: miR24–3p, miR181a-5p, miR146a-5p, and miR382–5p were significantly down-regulated in sera from PTC patients compared to healthy controls; three exosomal miRNAs: miR181a-5p, miR376a-3p, and miR382–5p were significantly down-regulated in sera from metastatic PTC (66). After a multiphase study demonstrating that miR-346, miR-10a-5p, and miR-34a-5p were significantly upregulated in PTC plasma samples relative to healthy controls, these three miRNAs also distinguished PTC from NG (142). Exosomal miR-148a-3p is significantly down-regulated in serum in DTC patients compared to benign tumors and healthy controls and correlates with malignant features of DTC such as tumor size and stage, and is a sensitive biomarker for DTC (143). Exosomal ncRNAs can also be used to differentiate between different types of TC. MiRNA-21 is overexpressed in plasma exosomes from patients with PTC versus benign tumors, whereas miRNA-21 helps to differentiate between benign tumors and follicular TCs (144). As a highly aggressive class of TCs, the levels of miR-210–3p in cancer cell lines and exosomes are significantly up-regulated in response to hypoxia-induced hypoxia, which is usually found in highly proliferative tumors, and thus miR-210–3p may be a marker of ATC hypoxia (145). In addition, exosomes hsacirc_007293, hsacirc_031752, and hsacirc_020135 were found to be up-regulated in serum of patients with PTC compared to NG, and may serve as biomarkers for diagnosis, prognosis, and treatment (146). Compared with normal individuals and benign thyroid tumors, the levels of exosomes hsa_circ_0082002 and hsa_circ_0003863 in PTC are upregulated. And higher levels of exosomes hsa_circ_0082002 and hsa_circ_0003863 are positively correlated with lymph node metastasis and vascular invasion of PTC (147).

Poor prognosis of TC is associated with recurrent metastasis after treatment, and liquid biopsy can also predict the clinical course of TC by ncRNA. As previously described, exosomal miR-485–3p from plasma overexpression correlates with advanced clinical stage and lymph node metastasis, and thus it can be used as a biomarker to differentiate between high-risk and low-risk PTC (65). By bioinformatics analysis, FN1, NMU, CHRDL1, GNAI1, ITGA2, GNA14, and AVPR1A were the seven genes closely associated with TC prognosis (148). ITGA2 is a direct target of miR-16, and downregulation of miR-16 leads to overexpression of ITGA2 (149). And exosomal miR-16 is downregulated in plasma of hepatocellular carcinoma patients and is a promising diagnostic, prognostic marker (150). MiR-29a acts as an upstream molecule of oncogenes to exert tumor-suppressive effects in both hepatocellular carcinoma and pancreatic cancer (151, 152). Its serum exosome miR-29a was similarly decreased in PTC, and down-regulation of miR-29a expression in PTC was associated with a high risk of recurrence in PTC patients, significantly correlating with poorer clinical variables and shorter survival (153). There was a statistically significant correlation between the high expression of miR-221–3p in plasma exosomes and tumor size, TNM stage, and lymphatic invasion; therefore, the expression level of miR-221–3p in plasma may be associated with the prognosis of PTC (83). The search for upstream exosomal ncRNAs about important oncogenes is subject to further development, in addition to liquid biopsies as biomarkers for therapy.

### 4.2 Liquid biopsy of exosomal ncRNAs RNA in TC

Given the rising incidence of overdiagnosis linked to thyroid cancer (TC) screening, identifying reliable biomarkers that can improve diagnostic accuracy is crucial (154). NcRNAs may serve as potential tools in this context, utilizing various detection methods that exhibit heterogeneity, including quantitative real-time PCR (qRT-PCR), small RNA sequencing, and miRNA sequencing analysis (155). Next-generation sequencing (NGS), also known as high-throughput sequencing (HTS), offers significant advantages such as cost-effectiveness and portability. With the maturation of this technology, its application in clinical practice is expected to become more widespread (155). Furthermore, the use of multiple biomarkers from the same class or a combination of various ncRNAs can enhance diagnostic precision. For example, a recent study developed a cyclic sEV miRNA (CirsEV miR) classifier using qRT-PCR technology, incorporating five miRNAs: miR-432–5p, miR-127–3p, miR-223–5p, miR-146a-5p, and miR-151a-3p. This classifier demonstrated an area under the curve (AUC) of 0.924 in the training set and 0.844 in the validation set, effectively identifying follicular thyroid carcinoma (FTC) patients (156). However, liquid biopsies, typically sourced from serum and plasma, may contain a mixture of exosomes from various cell types, both healthy and diseased. To address this, research has led to the development of a tissue chip device that isolates disease-specific extracellular vesicles from different thyroid pathological tissues maintained on the device. This innovation allows for a more targeted exploration of TC biomarkers, focusing on specific tissues of interest and studying the miRNA profiles of extracellular vesicles in benign conditions, Graves’ disease, and PTC (157). Screening for thyroid cancer using liquid biopsies that detect ncRNAs is highly promising. Next-generation sequencing (NGS) holds significant potential for the practical detection and sequencing of these genetic materials in future applications.

### 4.3 Potential applications in anti-TC therapy

Exosomes can be used to deliver drugs, molecules to treat TC, exosomes are a natural nanocarrier released by cells (including cancer cells), which have the advantage of low immunogenicity and cytotoxicity compared to viral vectors, and have the ability to be transported farther compared to liposomes (**Figure 4**) (158). MSC-derived exosomal miRNA-152 can then inhibit TC cell proliferation and metastasis by interacting with DPP4 (71). Exosomal mimics from NK cells also exhibited high anti-TC activity (114). Exosomes containing SCD-1 siRNA are able to promote ATC cell death by regulating fatty acid metabolism and reactive oxygen species levels (159). With respect to ATC, a recent study developed a multifunctional exosome delivery platform that combines internal irradiation and chemotherapy with iRGD-targeted exosomes as the delivery vehicle, which is capable of efficiently and accurately co-delivering 131I and Dox to ATC cells (160). Exosomal ncRNAs can not only directly target cancer cells, but also regulate TC progression by forming an anti-tumor microenvironment. For example, the clinical stage exosome product exoSTING can selectively target antigen-presenting cells (APC) in the tumor microenvironment to enhance CDN activity and stimulate anti-tumor immune response (161).

Exosomes can not only deliver various ncRNAs, but also natural compounds of plant origin, synthetic agents, siRNAs, and CRISPR/Cas9 can be used for tumor therapy via exosomes. Clinical experiments on the molecular profiling of exosomes are currently underway, which will inevitably provide a new pathway for precise and personalized tumor therapy in the future. Tumor heterogeneity is the biggest obstacle to all kinds of personalized immunotherapy, and the diversity of the tumor microenvironment is reflected in different cancers, different patients, and different periods of time (162). The latest methods of cancer organoid can better simulate tumor heterogeneity using organoids with reproducibility and accuracy. Cancer organoid biobanks have already been established for breast cancer, colorectal cancer and other cancers (163). However, cancer-like organs about TC still need to be researched, and the improvement of cancer-like organ models and precision tumor therapy will promote each other’s development, which will not be recounted here. Predictably, the co-development of exosomes and cancer-like organs will play an important role in the field of precision tumor therapy in the future.

To date, most exosomes and exosomal ncRNAs secreted from tumor cells and other cells derived from the tumor microenvironment play oncogenic roles in TC. Exosomes and exosomal ncRNAs, as important regulators of complex communication in the tumor microenvironment and TC, profoundly affect cancer proliferation, metastasis, angiogenesis, immune editing, and cell death through the tumor microenvironment. Therefore, drugs targeting exosomes and exosomal ncRNAs are an emerging therapeutic approach.

## 5 Methods of literature search

We conducted an extensive literature search on PubMed as of April 18, 2024. Keywords include exosomes, thyroid cancer. A total of 202 articles were found and screened for eligibility. The main criteria for inclusion include: (1) Include peer reviewed original research articles, comprehensive reviews, and meta-analyses; Prefer studies with robust methods, clear data analysis, and detailed reporting of results. (2) Studies should focus on exosomes and ncRNA, specifically miRNAs, lncRNAs, and circRNAs; Exclude studies that do not specifically address the composition and function of exosomal ncRNAs. (3) Only include studies that invest the role of exosomal ncRNAs in thyroid cancer, including their involvement in promotion, metathesis, EMT, angiogenesis, and immunoediting; Studies should provide insights into the mechanisms by which exosomal ncRNAs influence these oncogenic processes in thyroid cancer. (4) Include studies that explore the potential of exosomal ncRNAs as biomarkers for diagnosis, prognosis, or therapy response in thyroid cancer; Studies should discuss the stability of exosomal ncRNAs in biological fluids and their potential for non-invasive cancer monitoring. (5) Consider research discussing the therapeutic potential of targeting exosomal ncRNAs or using exosomes as delivery vehicles for therapy in thyroid cancer; Include studies that provide experimental evidence or theoretical discussions on the feasibility of these approaches. (6) Focus on studies published within the last 5–10 years to ensure the inclusion of the most recent and relevant data; Consider including landmark studies prior to this period if they have significantly contributed to the field. (7) Include studies from diverse geographical locations to cover a wide range of genetic backgrounds and environmental factors influencing thyroid cancer; Studies should ideally represent different population demographics to ensure broad applicability of findings. (8) Include studies published in English or with reliable English translations to ensure the accessibility of data for analysis; Ensure access to full-text articles to enable comprehensive review of methodologies and findings.

## 6 Conclusion

Exosomal ncRNAs play crucial roles in the development of thyroid cancer (TC) by mediating various molecular mechanisms and acting as key regulatory molecules within TC cells and the tumor microenvironment (TME). These ncRNAs facilitate oncogenic interactions, such as promoting the M2 phenotypic transformation of macrophages and enabling cancer-associated fibroblasts (CAFs) to enhance tumor cell proliferation and metastasis. This underscores the significance of exosomal ncRNAs in fostering communication between tumor cells and the TME. However, the specific functions of exosomal ncRNAs in TC, particularly in stromal cells, remain underexplored, presenting substantial opportunities for future research. This includes their roles in epithelial-mesenchymal transition (EMT), immune editing, angiogenesis, drug resistance, and radiotherapy sensitivity. Expanding and deepening our understanding of these mechanisms could offer new therapeutic avenues for TC treatment. Exosomal ncRNAs have also shown promise as biomarkers for tumor diagnosis, subtyping, treatment, and prognosis due to the low invasiveness and convenience of exosome examination, which compares favorably to other liquid biopsies like circulating DNA and tumor cells.

Yet, challenges remain in the clinical application of exosomal therapies, including issues with quality control, efficacy, safety, and standardized methods for exosome isolation. Furthermore, factors such as patient demographics and clinical characteristics contribute to tumor heterogeneity and may influence the types and contents of exosomal ncRNAs. At this stage, there is still a long way to go before exosomal ncRNAs can really enter the clinic from the experimental stage. Therefore, as the research on the role and mechanism of exosomal ncRNAs becomes broader and deeper, the diagnosis of TC will be more comprehensive and better, and the personalized targeted therapy for different patients will have a chance to become a reality.

Although most of the focus of our discussion on thyroid cancer is on molecular and genetic aspects, macro factors such as lifestyle also play a crucial role. Research has shown that lifestyle choices, including diet, exercise, and sleep, play a crucial role in regulating immune responses and potentially reducing the risk of thyroid cancer. Emphasizing the nutritional habits of a diet rich in antioxidants and omega-3 fatty acids may help reduce inflammation, thereby lowering the risk of thyroid cancer (164). In addition, regular physical activity and maintaining high-quality sleep are related to improving immune function, which can prevent the progression of thyroid tumors (165, 166). Cognitive participation and overall lifestyle balance can also help reduce systemic inflammation and improve health outcomes, which may affect thyroid health (167). Understanding the complex interactions between these lifestyle factors and their collective impact on thyroid tumors is crucial for developing effective prevention strategies and promoting successful aging (168, 169). In future studies, exploring how lifestyle factors regulate exosomal pathways or interact with ncRNAs could uncover new directions for thyroid cancer research, potentially leading to innovative prevention strategies and personalized therapies.

## Author contributions

WS: Writing – review & editing. CJ: Writing – original draft. QL: Writing – original draft. NW: Writing – original draft. RH: Writing – review & editing. GJ: Writing – original draft. YY: Writing – original draft.
